# Obesity in the Liver Transplant Setting

**DOI:** 10.3390/nu11112552

**Published:** 2019-10-23

**Authors:** Carlos Moctezuma-Velazquez, Ernesto Márquez-Guillén, Aldo Torre

**Affiliations:** Division of Hepatology and Liver Transplant, Department of Gastroenterology, Instituto Nacional de Ciencias Médicas y Nutrición “Salvador Zubirán”, Mexico City 14080, Mexico; mocmocte@hotmail.com (C.M.-V.); ernesto47mx@hotmail.com (E.M.-G.)

**Keywords:** body composition, obesity, liver transplantation, liver cirrhosis

## Abstract

The obesity epidemic has resulted in an increased prevalence of obesity in liver transplant (LT) candidates and in non-alcoholic fatty liver disease (NAFLD) becoming the fastest growing indication for LT. LT teams will be dealing with obesity in the coming years, and it is necessary for them to recognize some key aspects surrounding the LT in obese patients. Obesity by itself should not be considered a contraindication for LT, but it should make LT teams pay special attention to cardiovascular risk assessment, in order to properly select candidates for LT. Obese patients may be at increased risk of perioperative respiratory and infectious complications, and it is necessary to establish preventive strategies. Data on patient and graft survival after LT are controversial and scarce, especially for long-term outcomes, but morbid obesity may adversely affect these outcomes, particularly in NAFLD. The backbone of obesity treatment should be diet and exercise, whilst being careful not to precipitate or worsen frailty and sarcopenia. Bariatric surgery is an alternative for treatment of obesity, and the ideal timing regarding LT is still unknown. Sleeve gastrectomy is probably the procedure that has the best evidence in LT because it offers a good balance between safety and efficacy.

## 1. Introduction

**Figure 1:**
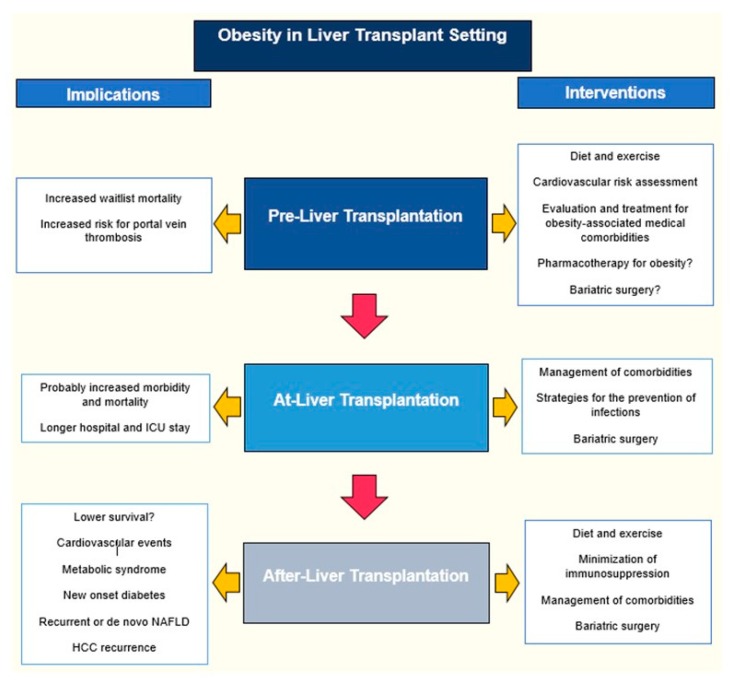
The impact of obesity in the liver transplant (LT) setting. This flowchart illustrates the key elements to consider when assessing a patient with obesity in the context of LT.

The obesity epidemic has resulted in an increased prevalence of obesity in liver transplant (LT) candidates, in non-alcoholic fatty liver disease (NAFLD) becoming the fastest growing indication for LT [1], and in hepatocellular carcinoma (HCC) becoming one of the leading indications for LT [2,3]. There is an estimated lag of around nine years between the increase in the prevalence of obesity and its impact on the number of patients listed for LT secondary to NAFLD. In the United States, obesity has increased by 45% from 2000 to 2014, and in the same lapse, NAFLD-related annual waitlist additions went from 391 to 1605, and these are expected to increase by 55% between 2016 and 2030 [4]. In the case of Europe, based on the European Liver Transplant Registry (ELTR) database, NAFLD as the underlying etiology for LT increased from 1.2% in 2002 to 8.4% in 2016 [5]. The 2017 Organ Procurement and Transplantation Network/Scientific Registry of Transplant Recipients (OPTN/SRTR) LT report revealed that 38.5% and 15% of LT recipients were obese and morbidly obese, respectively, compared to 20.6% and 12.4% during 2007 [6]. Based on this, LT teams will be dealing with obesity more frequently in the coming years; therefore, the aim of this review is to summarize key points regarding obesity pre, during, and after LT. Different body mass index (BMI) cutoff points have been used to define overweight and obesity in the LT patients, and to avoid confusion, we will use the following thresholds, unless specified otherwise: Normal weight (BMI 18.5−24.9 kg/m^2^), overweight (BMI 25–29.9 kg/m^2^), grade I obesity (BMI 30–34.9 kg/m^2^), grade II or severe obesity (BMI 35–39.9 kg/m^2^), and morbid obesity (BMI > 40 kg/m^2^). Figure 1 summarizes the key elements to consider in the evaluation of a patient with obesity in the context of LT.

## 2. Obesity Before Liver Transplantation

### 2.1. How Should Obesity be Defined in Patients with Cirrhosis Considered for LT?

The first obstacle is to define obesity in this population. Elamparast et al. recently summarized the most commonly used definitions in this group of patients [7]:

- BMI ≥ 30 kg/m^2^ (Asian population: ≥25 kg/m^2^);
- Percentage of fat mass ≥28% in men and ≥40% in women;
- Waist circumference ˃102 cm in men and ˃88 cm in women (Asian population: A waist circumference ˃90 cm in men and ˃80 cm in women);
- Visceral fat area ≥100 cm^2^ on abdominal computed tomography.

The use of BMI has several limitations in patients with decompensated cirrhosis [8], as reflected by the fact that sarcopenia outperforms BMI as a predictor of outcomes after LT [9]. First of all, a high BMI may be due to fluid overload/ascites, and not obesity per se, as exemplified in a study in which adjusting BMI for the liters of ascites drained at the time of LT resulted in moving one-fifth of patients from one BMI category to a lower one [10]. Also, BMI gives no information about body composition: It cannot tell the difference between muscle, subcutaneous, or visceral adipose tissue, which have different implications. Studies looking at body composition on cross-sectional imaging have found that sarcopenia and low subcutaneous adipose tissue, but not BMI, are associated with mortality in male and female patients with cirrhosis, respectively [11]. Excessive visceral fat when associated with sarcopenia, a term known as sarcopenic obesity, is also associated with increased mortality [12,13,14]. In the case of living donor liver transplant (LDLT) patients, sarcopenia, myosteatosis, and the visceral-to-subcutaneous-adipose-tissue ratio, but not visceral or subcutaneous tissue on their own, have been associated with mortality [15]. This reinforces the fact that more than the size of a patient, which is what the BMI stands for, it is the composition that matters. For the same reasons, waist circumference is not the best method to assess obesity in these patients. Trying to circumvent some of these limitations, the European Association for the Study of the Liver guidelines for nutrition in patients with cirrhosis recommend to adjust the BMI for the confounding effect of fluid retention (i.e., dry-BMI) [16], though there is no perfect correlation between BMI and ascites [17], and in fact, BMI corrected for ascites drained at the time of LT failed to predict relevant outcomes after LT [10,18]. Likewise, a modified BMI calculated by multiplying BMI (kg/m^2^) by serum albumin (g/L) with the intention of compensating for volume overload was found of no use to predict clinical outcomes [18].

In conclusion, when available, we suggest obesity be defined according to the visceral fat area on body composition analysis, especially in the research setting, because the impact of obesity on LT may be modified by the coexistence of sarcopenia, myosteatosis, and/or low subcutaneous adipose tissue. 

### 2.2. Should Morbidly Obese Patients be Listed for LT?

Some LT programs consider morbid obesity a relative contraindication for LT based on the latest recommendations from the American Association for the Study of the Liver [19,20]. However, evidence evaluating the role of obesity on outcomes after LT is conflicting. When needed, patients with severe and morbid obesity do benefit from LT, meaning they live longer than they would do without it [21], and LT in these patients strays far away from being futile, with five-year survival rates well above 50% [22]. Evidence so far does not support using a strict BMI cutoff to deny someone candidacy for LT, however obesity is frequently associated with other conditions that may be contraindications for LT such as cardiovascular disease, cancer, renal dysfunction, and pulmonary hypertension. Also, obesity is a risk factor for NAFLD, which though traditionally considered to have similar post-LT outcomes to those of other etiologies of liver disease [23], may in fact have lower one-year survival due to cardiovascular and cerebrovascular diseases [24]. In summary, more than obesity itself, it is the sum of the comorbidities of the individual patient that determines LT outcomes [25] and as such, morbid obesity should not be considered on its own a contraindication for LT, but in turn should trigger a rigorous pre-LT assessment to carefully select potential recipients [23], as discussed below. 

### 2.3. How does Obesity Impact LT Assessment?

Obesity is an independent risk factor for cardiovascular disease and usually coexists with other risk factors; hence, special attention should be paid regarding the pre-LT cardiovascular workup and risk assessment. There is an extensive debate of the optimal cardiac evaluation of LT candidates, which is outside the scope of this review [26], but all patients require electrocardiography and echocardiography on a routine basis [22], and there should be a low threshold to trigger different modalities of cardiac testing in order to uncover asymptomatic ischemic heart disease. As per the American Heart Association recommendations, patients with additional risk factors such as diabetes, left ventricular hypertrophy, prior cardiovascular disease, age greater than 60, smoking, hypertension, and/or dyslipidemia should be considered for further testing [27]. Noninvasive stress testing and noninvasive imaging tools such as coronary artery calcium score may be useful in triaging patients for invasive methods such as coronary angiography [23]. Of note, a systematic review that evaluated risk factors for adverse cardiovascular outcomes in LT patients found age and history of cardiac disease, but not BMI, as predictors of cardiovascular events post-LT [26]. 

As part of the LT assessment, special emphasis should be placed on following conventional oncologic screening recommendations because obesity is an independent risk factor for multiple malignancies. 

There is an additional obstacle in the setting of LDLT assessment: Achieving an acceptable graft weight/recipient weight (GWRW) ratio. Patients that are rejected from LDLT programs due to an unacceptable GWRW ratio have higher BMIs. Moreover, in order to achieve an adequate GWRW, obese patients need livers from donors with higher BMIs, who may be at higher risk of being turned down because of NAFLD [28]. 

### 2.4. How does Obesity Impact Patients while on the Waiting List?

Patients with morbid obesity have a higher risk of dying while on the waiting list [21], and this may be due to multiple factors. Obesity per se may worsen the natural history of cirrhosis, as shown in a subanalysis of the pivotal trial that compared timolol versus placebo in the prevention of esophageal varices, in which BMI was found to be an independent predictor of the first clinical decompensation and obesity was associated with a lack of decrease in the hepatic venous pressure gradient (HVPG) at one-year follow up. It is possible that leptin and the pro-inflammatory milieu seen in the obese increase intrahepatic vascular resistance, worsening portal hypertension [29,30,31]. Concomitant comorbidities may have more prognostic impact than obesity itself while on the waiting list. For example, in a retrospective analysis of the SRTR of 25,647 waitlisted patients, covariate adjusted mortality (i.e., age, gender, race, ascites, etiology, and the model for end-stage liver disease (MELD)) while on the waiting list was not different in patients with overweight, obesity, or morbid obesity, when compared to normal weight patients [32]. Similarly, a study looking at data from the Hepatitis C antiviral long-term treatment against cirrhosis (HALT-C) trial, which included patients with advanced fibrosis as well as compensated cirrhosis, found that BMI was associated with decompensation and death on univariate analysis, but not after adjusting for diabetes and insulin resistance [33]. Finally, severe and morbid obesity decreased the odds of receiving a MELD exception and of being transplanted, and increased the chance of being turned down for an organ [34], resulting in a higher waitlist dropout and death [35]. Part of this disadvantage at the time of allocation may be because morbidly obese patients are usually sicker, with higher MELD scores, and at higher risk of being in the intensive care unit (ICU) while on the waiting list [1,18].

Once obese patients are listed, it is crucial to do regular imaging to detect portal vein thrombosis in a timely fashion because obesity is a risk factor for this complication [36].

On the other side of the coin, the obesity paradox, where obese patients have better outcomes than patients with normal BMI, may also be true in cirrhosis. Obesity in inpatients with underlying cirrhosis has been associated with lower inpatient mortality, presumably because obesity provides some degree of nutritional reserve that allows patients to withstand a critical illness [37].

### 2.5. How should Overweight/Obesity be Managed in Patients Listed for LT?

Treatment of obesity is not straightforward because most of these patients are decompensated and uncontrolled weight loss may lead to worsening frailty and sarcopenia. Treatment of obesity may have theoretical benefits, such as decreasing the risk of decompensation and HCC, improving fibrosis in patients with NAFLD cirrhosis, improving candidacy for LT, and improving the control of associated comorbidities [38]. Nonetheless, there is no evidence to support losing weight while on the waiting list to improve outcomes. 

#### 2.5.1. Diet and Exercise

Diet and exercise should be the cornerstone of the treatment strategy, and in some cases, the only therapy needed. In a retrospective study of 49 patients who had severe obesity at the time of LT assessment, 36 of them were able to achieve a BMI < 35 kg/m^2^ with a noninvasive multidisciplinary weight-loss program and then successfully undergo LT. Three years after the LT, 22% of them were able to maintain a BMI < 35 kg/m^2^ [39,40]. 

Intentional weight loss in patients with obesity and decompensated cirrhosis needs to be carefully tailored and supervised. As stated by the experts of the International Society for Hepatic Encephalopathy and Nitrogen Metabolism Consensus, weight loss in this group of patients should consider reducing calories from carbohydrate and fat content while maintaining a high protein intake (1.2–1.5 g/kg of ideal body weight). Target daily energy intake should be 20–25 and 25–35 kcal/kg of ideal body weight in patients with morbid obesity and obesity, respectively [41], avoiding diets with less than 1000 calories daily [42,43], though there are some case series describing very-low-calorie diets in patients with end-stage liver disease [44]. To reduce muscle breakdown, patients should avoid prolonged fasting periods (i.e., more than 4–6 h) by frequent meals and by providing a late night snack [7].

There is a general misconception that patients with cirrhosis cannot rely on exercise to achieve weight loss. Two things should be emphasized in this scenario. First, the increase in HVPG that occurs in patients with cirrhosis and portal hypertension during physical exercise can be prevented with propranolol. Therefore, before patients with cirrhosis embark into an exercise program, it is imperative to determine as per current guidelines if they have an indication for prophylaxis against variceal hemorrhage [45,46]. Second, patients doing exercise should have an adequate intake of proteins and nutrients to promote reduction of fat mass, but not worsening of sarcopenia. Sustained exercise in patients with compensated cirrhosis is safe and provides important benefits such as reductions in HVPG, and improvement of fitness and of their nutritional status [47]. Easy interventions such as walking 5000 or more steps a day protect against sarcopenia [48]. A pilot study of supervised aerobic exercise (cycle ergometer three days/week) in patients with cirrhosis, most of them with clinically significant portal hypertension (CSPH), was safe and resulted in increased peak exercise oxygen uptake and muscle mass, and improvement in fatigue and patient´s self-perceived health status [49]. Another pilot study showed how a 12-week supervised moderate exercise (60–70% of maximum heart rate) program in patients with compensated cirrhosis resulted in improved performance, muscle mass, and quality of life [50]. The SportDiet study demonstrated that diet and exercise are feasible, safe, and effective strategies to lose weight in patients with compensated cirrhosis, portal hypertension, and a BMI > 26 kg/m^2^. In this study, 60 cirrhotic patients with overweight and portal hypertension (i.e., HVPG > 5 mmHg) were enrolled in a 16-week program of diet (hypocaloric, normoproteic) and exercise (60 min/week of supervised physical activity). Diet and exercise resulted in weight loss of at least 5% and HVPG reduction of at least 10% in 5% and 42% patients, respectively [30]. In the case of patients with decompensation and intense activation of the renin-aldosterone and sympathetic nervous systems, one should be cautious, as moderate exercise may impair renal function. However, a small study showed that a personalized adapted physical activity program is safe in decompensated patients awaiting LT, the problem being that these programs need to be strictly supervised and require individualized planning, which is time-consuming [51]. 

In summary, weight loss can be achieved through moderate caloric restriction as long as there is an adequate intake of proteins. In patients with compensated cirrhosis, it is safe to do moderate physical activity once an adequate prophylaxis for variceal bleeding has been established. More evidence is needed to support the safety and efficacy of exercise in decompensated patients, but personalized adapted physical activity programs seem feasible and promising, though there is no evidence showing that exercise and weight loss lead to improved outcomes while on the waiting list or after LT.

#### 2.5.2. Pharmacologic Treatment

Information about the safety and efficacy of anti-obesity medications in patients with cirrhosis is very limited [38]. According to the prescribing label, Orlistat, a gastric and pancreatic lipase inhibitor, can be used in patients with liver impairment without need for dose adjustment. Lorcaserin, a selective agonist of 5-hydroxytryptamine receptor 2C, does not need drug adjustment in patients with Child A/B cirrhosis, and has not been studied in Child C patients. It has not been formally studied in patients with cirrhosis, but there is a case report of a patient with decompensated cirrhosis and morbid obesity that achieved significant weight loss with Lorcaserin [52]. Phentermine/Topiramate is an FDA approved weight loss combination that needs no dose adjustment for compensated cirrhosis; the maximum dose is 7.5 mg/46 mg qd in patients with Child B cirrhosis, and it has not been studied in Child C patients. The combination of Naltrexone/Bupropion is also approved for weight loss but has not been studied in patients with cirrhosis; in the case of hepatic impairment, the maximum recommended daily dose is 8 mg/90 mg. Finally, Liraglutide, a glucagon-like peptide-1 receptor agonist, though not formally studied for treatment of obesity in patients with cirrhosis, does not need dose adjustment, and could be a useful tool to treat obesity in patients with cirrhosis, but more evidence is needed [38]. In summary, there is no evidence of the use of anti-obesity medications in patients with cirrhosis, but Liraglutide and Orlistat seem safe alternatives that could be used in patients with compensated cirrhosis. Importantly, patients on Orlistat should receive supplements with liposoluble vitamins. There is no evidence to support the role of these medications in decompensated cirrhosis and cannot be recommended. 

#### 2.5.3. Bariatric Surgery in the Pre-LT Setting

The best timing of bariatric surgery (BS) with respect to LT is a matter of debate and should be decided on a case by case basis. Table 1 shows the pros and cons of performing BS pre, during, or post-LT. Overall, bariatric-surgery performed in perfectly compensated cirrhosis is feasible, achieving outcomes that are very close to those reported in the general population [53,54,55,56], with no prohibitive morbidity or mortality. Mosko et al. compared in-hospital mortality and length-of-stay of 674,900 patients that underwent BS between 1998 and 2007, of whom 3888 and 62 had compensated and decompensated cirrhosis, respectively. Mortality rates were higher in patients with decompensated cirrhosis, followed by compensated cirrhosis, when compared with non-cirrhotic patients (16.3%, 0.9%, 0.3%, respectively), and the same happened with hospital length of stay (6.7, 4.4, 3.2 days, respectively). On multivariate analysis, cirrhosis was confirmed as an independent predictor of mortality [57]. A meta-analysis that evaluated the role of BS in LT included 11 studies and 56 patients: Two studies (26 patients) evaluated BS pre, two studies (8 patients) during, and seven studies (22 patients) post-LT. The most commonly performed procedure was sleeve gastrectomy (SG), followed by Roux-en-Y gastric bypass (RYGB). Six patients died: Three while on the waiting list, after SG, and three patients that underwent BS after LT. Importantly, approximately 12% of patients required a reintervention for complications, including two staple-line leaks after SG. In general, there was improvement of liver tests after BS, and the percentage of excess weight loss was similar to that described in the non-LT setting, but morbidity rate was higher [58].

Potential benefits of BS are being listed for LT in places where a specific BMI cutoff point precludes candidacy to LT, and improving the control of comorbidities associated with obesity, including NAFLD, in which even regression of fibrosis can be achieved [56,59,60,61,62]. Amongst the risks of BS in the pre-LT setting is that sarcopenia, a predictor of mortality in cirrhosis, may worsen [63,64], and patients may become malnourished. Hence, the long-term potential complications after BS in patients with underlying cirrhosis should also be emphasized. Another potential risk is precipitating liver failure, which is infrequent nowadays, and was mostly commonly seen in the early days of BS, when procedures bypassed most of the small bowel, leading to malnutrition and liver failure in a significant number of cases. A recent systematic review compiled 32 patients that underwent LT because of liver failure attributed to BS; the most common procedures were jejunoileal bypass (50%), and bilio-pancreatic diversion (44%), while there was a single case of RYGB, and there were no cases attributed to SG [65,66].

In conclusion, BS in the pre-LT setting should be reserved for patients with compensated cirrhosis and no evidence of CSPH, though the use of transjugular intrahepatic portosystemic shunts has been explored by some to decrease portal hypertension and the risk of perioperative complications [61]. Pre-LT patients are usually too decompensated to safely undergo a bariatric procedure, but in case of pursuing BS pre-LT, experts recommend to do it at least one year before LT is contemplated to achieve a stable weight, and to allow for improvement of obesity associated comorbidities [67]. In our opinion, BS should only be considered in the context of LT when BMI is an obstacle for getting listed, and it should be preferentially done laparoscopically and in centers with expertise in this area. SG seems to be the best alternative [53,60] because it has a lower perioperative risk, lower risk of nutritional status worsening, it allows one to preserve access to the biliary tree via endoscopy, it does not interfere with immunosuppression pharmacokinetics, and it does not restrict access to the stomach in case there is gastric variceal bleeding [59]. Six-month and one-year weight loss with SG in these patients is around 18–28% and 25–34%, respectively [62] (Reibibo L, 2014). Table 2 shows the potential advantages and disadvantages of the different procedures. 

#### 2.5.4. Use of Intragastric Balloons for Obesity Management

The use of intragastric balloons sounds promising for patients with morbid obesity and decompensated cirrhosis that are deemed ineligible for LT due to their BMI, but that are too sick to safely undergo a BS, but more evi–dence is needed. Small studies reporting outcomes in patients with decompensated cirrhosis and CSPH [68] show an acceptable safety profile. However, the presence of gastric varices, large esophageal varices, or severe portal hypertensive gastropathy, all of which are commonly found in the decompensated patient, are contraindications for this procedure [69,70]. 

### 2.6. Obesity-Related Comorbidities in the Pre-LT Setting

Obesity, as part of the metabolic syndrome, is usually associated with other comorbidities that may explain part of the association between obesity and waitlist mortality, and between obesity and increased post-LT mortality, as will be explained later. Therefore, in order to achieve satisfactory long-term outcomes after LT, it is important to identify, assess, and treat in a timely fashion all cofactors that increase cardiovascular risk; special attention must be paid to the adequate control of dyslipidemia, hypertension, and diabetes.

## 3. Obesity at the Time of Liver Transplantation

### 3.1. Are Obese Patients at Increased Risk of Peri-Operative Morbidity and Mortality? 

The answer is not clear, and there is considerable debate on this topic because evidence is conflicting. Some studies have found that these patients have increased peri-operative morbidity and mortality [71,72,73,74,75,76,77], whereas others have not [78,79,80,81,82,83]. Discordance between studies may be because some of them have studied obesity, whereas others have focused on morbid obesity. In addition, comorbid conditions associated with obesity such as diabetes may have important implications on outcomes, underscoring the need to adjust for those cofactors. Regretfully, only the few following studies have controlled for confounders. A study performed in a retrospective cohort of 202 consecutive patients that underwent LT looked at the additive effect of pre-LT cardiovascular risk factors, including obesity, and post-LT morbidity, and found obesity to be a risk factor for postoperative complications in the presence of concomitant diabetes. These complications were infections (wound infection, bacteremia, pneumonia), cardiovascular events (arrhythmia, infarction), and respiratory events (pneumonia, effusion). As a result, patients with concomitant obesity and diabetes had longer hospital stays [84]. Another study in 617 patients undergoing LT also found that obesity in the presence of diabetes, but not on its own, was associated with longer hospital stays [85]. An analysis of the Nationwide Inpatient Sample of the United States database of 46,509 patients that underwent LT, including 818 that had morbid obesity, showed after propensity score matching that complication rates were equally distributed between the two groups, except for respiratory complications, which were more frequent in the morbidly obese [86]. 

A meta-analysis that specifically addressed post-operative complications of LT in patients with obesity found grade I obesity to be associated with post-operative cardiopulmonary complications, 30-day mortality, and length of hospital stay; morbidly obese patients also had higher 30-day mortality [87]. Another finding that has been consistent across different studies is that obesity increases the risk of infectious complications, particularly of wound infections [72,73,75,76,88].

All in all, though data are controversial, obesity may be a risk factor for increased perioperative morbidity, especially in the presence of coexistent diabetes, and mainly due to infectious and respiratory complications, which results in longer hospital stays [18,84]. Therefore, it is imperative to establish pre- and post-operative measures to prevent post-LT complications. These measures should be focused, but not limited, to: Active monitoring for infections, including wound infection, antibiotic prophylaxis when indicated, exhaustive pre-LT cardiac evaluation, active monitoring post-LT, careful fluid management, oral chlorhexidine, and incentive spirometry [18,84]. 

### 3.2. Bariatric Surgery at the Time of LT

In addition to promoting weight loss, BS at the time of LT helps control comorbid conditions that are usually exacerbated after LT due to immunosuppression, such as diabetes, dyslipidemia, and hypertension. Heimbach et al. described their experience with 13 patients that underwent simultaneous LT and SG. There were no deaths, but two complications: Leak from the gastric staple line and excessive weight loss in one patient. After LT, weight gain to BMI > 35 kg/m^2^, diabetes, and steatosis, developed in 60%, 34%, and 20% of patients that only had had LT, respectively, versus 0% in patients in the BS group. After three years, the percentage of total body weight loss was significantly higher in the SG group (35% vs. 4%) [39,40]. Though evidence is limited, SG at the time of LT has shown an acceptable safety profile and good outcomes in terms of weight loss [53] and can be considered to simultaneously address end-stage liver disease and severe or morbid obesity in centers with expertise in this area. Table 1 and Table 2 show the advantages and disadvantages of performing BS at the time of LT, the risks, and benefits of each approach.

### 3.3. Obesity and Organ Allocation

As the prevalence of obesity increases worldwide, the number of steatotic grafts is expected to increase, jeopardizing the rate of donor graft utilization [89]. Grafts with mild macrovesicular steatosis may be used without major concerns, whereas those with severe steatosis should not be used due to the increased risk of primary non-function and dysfunction secondary to ischemia/reperfusion injury. Due to the organ shortage, grafts with moderate steatosis have been used with acceptable outcomes as long as other extended-criteria factors are avoided [90]. There is currently ongoing research of different pharmacological approaches and the use of machine perfusion methods to optimize marginal grafts, including those with steatosis, with promising results, but nevertheless, more evidence is needed [91]. Another factor to consider in organ allocation is that adverse effects of using liver grafts with prolonged cold ischemia are potentiated in the presence of obesity in the recipient [92].

## 4. Obesity After Liver Transplantation

### 4.1. What is the Impact of Pre-LT BMI on Post-LT Patient and Graft Survival?

Whether obesity affects patient or graft survival after LT is still a matter of debate. Despite obesity being a well-known risk factor for cardiovascular events and cancer, which are two important causes of death in patients after LT [22], the association between pre-LT obesity and dismal outcomes is controversial. This may be due to selection bias, because obese patients usually undergo an extensive cardiac workup before getting listed, and because most programs emphasize oncologic screening after LT [22].

There are several studies that have found no effect of obesity, severe obesity, or morbid obesity, on short- and mid-term patient or graft survival [21,32,72,74,93]. Nair et al. published one of the first studies to link obesity with poor outcomes after LT, and it was based on his publication that the American Association for the Study of the Liver guidelines for LT on 2005 stated morbid obesity as a contraindication for LT [20,71,94]. In their study, based on the United Network for Organ Sharing database, they analyzed 18,172 LTs performed on the pre-MELD era and found that the severely obese had lower five-year survival, and the morbidly obese had lower one-year, two-year, and five-year survival. Excess mortality was attributed to cardiovascular events. One of the cardinal limitations of this study is that BMI was not adjusted for ascites. After the study by Nair, there have been several others linking obesity, specifically severe and morbid obesity, with decreased survival after LT [1,95,96]. Of note, most studies that have adjusted for concomitant comorbidities, and especially for diabetes, by either multivariate analysis [18,73,85,97,98,99,100] or through propensity score matching [86,101], have failed to show an independent role of obesity on patient´s outcomes. Whereas morbid obesity and severe obesity may have a negative impact on outcomes, overweight and mild obesity are associated with better survival [98,99,102].

There are data suggesting that the interaction between BMI and NAFLD should be considered when evaluating post-LT outcomes. A recent study that interrogated the ELTR database (2002–2016) to study the outcomes of patients with NAFLD undergoing LT found, through multivariate analysis, that morbid obesity, MELD > 23, and age were independent predictors of death. However, BMI was not corrected for ascites, which limits the interpretation of the findings given the operational definition of NAFLD cirrhosis that was used (i.e., cirrhosis of unknown origin in association with BMI > 30 kg/m^2^). Also, the multivariate analysis did not include other comorbidities that run along with NAFLD and that may influence survival such as the metabolic syndrome, coronary artery disease, or chronic kidney disease [5]. Another study looking at patients undergoing LT for NAFLD cirrhosis found that patients that died early after LT tended to be older, more obese, and with a higher frequency of diabetes and hypertension. In fact, patients that had all these four characteristics had a one-year survival of only 50%, but no multivariate analysis was performed to assess the specific role of obesity. Nonetheless, as the authors pinpointed, careful pre-LT assessment is warranted before offering LT to these high-risk patients [103]. 

At least three systematic reviews and meta-analyses have been performed looking at the role of pre-LT BMI on LT outcomes. The first of them is the meta-analysis by Saab et al. that comprised 13 studies involving 76,620 patients (72,212 non-obese and 2275 obese) and found no association between different thresholds of BMI (i.e., 25, 30, 35, 40) and mortality. Sensitivity analysis also showed no association in studies that adjusted BMI for ascites, but did find a lower survival in obese patients in a subgroup analysis according to etiology of liver disease [104]. However, the control group in this meta-analysis was criticized because it excluded overweight patients and included malnourished patients, which are known to have a poor prognosis. A second meta-analysis published by Barone et al. that took care of the limitations of the previous meta-analysis, found that patients with morbid obesity had a higher one-, two-, and five-year mortality rates [87]. Finally, the study by Beckmann et al. showed no differences in patient or graft survival rates at specific time points (e.g., 30-day, one-year, five-year) between obese and non-obese LT recipients. However, it was found that patients with obesity had shorter overall patient and graft survival, though there was marked heterogeneity between the studies, limiting the validity of the results. Also, no meta-regression was done. Importantly, a sub-analysis based on year of publication showed that the more recent the study, the longer the survival reported, reflecting improvement in LT care [105]. In this case, meta-analyses, though useful, are not capable of answering whether increased mortality seen in some studies is due to the comorbidities associated with obesity, or to obesity itself. 

In conclusion, due to the heterogeneity of the results, it is impossible to draw firm conclusions about whether obesity by itself is risk factor for poor LT outcomes. Nonetheless, as Thuluvath states, in real life, obesity comes as a full package [106], and therefore, to recapitulate the good outcomes seen in some studies, it is essential to do a thorough pre-LT assessment to offer LT only to those ideal obese patients [107]. Based on the more consistent findings on the different studies, morbid obesity, and probably severe obesity, may have some degree of impact on LT outcomes, particularly in patients with NAFLD cirrhosis and/or with diabetes. The role of overweight or mild obesity is even less clear, and they may even have protective effects. Most of the studies showing good outcomes in obese patients have evaluated short and mid-term outcomes, and there is some paucity of data on long-term survival, which may be affected by the burden of cardiovascular complications [108], so more data are needed regarding long-term follow-up.

### 4.2. What is the Impact of Pre-LT BMI on Other Post-LT Outcomes?

Pre-LT BMI and weight gain after LT, which is particularly accelerated during the first two years, are risk factors for development of metabolic syndrome, NAFLD, and new onset diabetes after transplant [105,109,110,111]. Therefore, weight should be closely monitored after LT, especially in those patients at increased risk of weight gain such as those who are older than 50, those with NAFLD as underlying liver disease, and those with pre-LT obesity [110,112,113,114]. 

Patients with obesity have a higher rate of comorbidities associated with the metabolic syndrome when compared with patients with lower BMI [86], with a subsequent higher cardiovascular risk. The CAR-OTL score was developed using pre-LT variables to predict the risk of cardiovascular disease within one year after LT. Though it still needs further validation, it may be used to identify patients at increased risk of this outcome in order to keep a strict control of the modifiable risk factors. Of note, BMI was not included in the model because it was not associated with adverse outcomes [115]. A small retrospective study of 170 patients that underwent LT also showed no difference in the rate of cardiovascular complications between obese and normal-weight recipients after a mean follow up of 5 years [116]. 

Pre-LT obesity is a risk factor for HCC recurrence. A study of 159 patients undergoing LT for HCC found that recurrence of HCC was twice as frequent in patients with overweight/obesity when compared with normal-weight individuals, and they had a shorter time-to-recurrence [117]. Similarly, in a study of 342 patients that underwent LT for HCC, an association was found between obesity, microvascular invasion, and poor survival [118], probably because adipokines promote angiogenesis. A study that evaluated the impact of skeletal muscle mass to visceral fat area ratio in LDLT recipients due to HCC found that a low ratio, but not BMI, was associated with a lower recurrence-free and overall survival [119,120] reinforcing that BMI may not be the best tool to define obesity.

The role of obesity as a risk-factor for vascular complications after LT has been controversial. However, a meta-analysis that pooled the results of six retrospective cohort studies found no association between obesity and vascular or biliary complications. The same results were obtained considering different BMI cutoff points, and when considering an ascites-adjusted BMI [121].

Finally, in terms of financial costs, a study in 700 LTs found that overweight and obesity had no impact on monetary costs [122], but obesity does seem to delay improvement in physical quality of life initially after LT [123]. 

### 4.3. How should Obesity be Treated after LT?

The implications and treatment of obesity after LT are outside the scope of this review, but in general, management should include the same steps as in other patients: Diet and exercise, pharmacologic therapy, and BS. Orlistat was evaluated in the post-LT setting and seems to be safe as long as immunosuppression levels are closely monitored [124], but there are no data regarding its efficacy. BS is also possible after LT, but may be more technically demanding, and associated with increased morbidity when compared with non-LT patients. It is reasonable to wait at least one year after the LT to reduce the risk of rejection from modifications of immunosuppression, and to reduce the risk of infectious complications associated with immunosuppression, which is highest during the first year [67]. Also, use of steroids has been associated with increased 30-day post-BS morbidity and mortality in the general population [125], and most patients will receive steroids at least during the first 3–6 months after LT. The safety and feasibility of SG has been well reported [126,127,128], and there are also reports of RYGB, intragastric balloon [129], and robot-assisted minimally invasive BS [130]. Laparoscopic adjustable gastric banding (LAGB) remains poorly explored in this setting, probably due to the potential for side effects in terms of infection of a foreign body in the context of a patient under immunosuppression. Considering the immunosuppression needed after LT, pharmacokinetic studies have shown that SG does not affect the kinetics of tacrolimus or mycophenolic acid, which is convenient. In opposition to this, the pharmacokinetics of tacrolimus, sirolimus, and mycophenolic acid do get significantly altered in the RYGB population, which can result in the need for higher doses [131,132]. Table 2 includes pros and cons of the different surgical techniques and of the endoscopic intragastric balloon as well (RYGB, SG, LAGB, endoscopic intragastric balloon).

### 4.4. How should Immunosuppression be Managed in the Obese Recipient?

Immunosuppressive agents are a risk factor for different metabolic side effects. Steroids and calcineurin inhibitors are more strongly associated with diabetes and hypertension, and mammalian target of rapamycin inhibitors with dyslipidemia. There is no specific immunosuppression scheme that has been shown to be effective in preventing weight gain after LT, but immunosuppression should be tailored to reduce as much as possible the risk of metabolic complications. As such, even if the use of steroids does not seem to impact weight gain [114,133,134], steroid-free protocols and calcineurin inhibitors minimization strategies should be considered to decrease the risk of metabolic complications, including new onset diabetes and hypercholesterolemia [113,135,136]. A post-hoc analysis of a randomized controlled trial that compared everolimus and reduced tacrolimus levels vs. regular tacrolimus dosing found that one-year and two-year weight gain was attenuated in the latter group, suggesting mammalian target of rapamycin inhibitors may be a promising alternative to prevent worsening of obesity [137].

## 5. Conclusions

In the following years, there will continue to be a shift in the frequency of the leading indications of LT, and NAFLD will become the number one indication. With obesity being so intimately linked with NAFLD, establishing strict BMI cutoff points as a contraindication for LT would probably lead to an unnecessary increase in the death rate of patients with liver cirrhosis, without necessarily improving overall survival after LT. Instead, obesity should be a trigger to do a rigorous pre-LT evaluation, with special focus on the cardiovascular risk assessment, in order to properly select candidates for LT. Evidence so far supports that patients with obesity may be at increased risk of morbidity surrounding LT, and it is necessary to establish preventive strategies. Regarding patient and graft survival, data are controversial, and there is a lack of evidence on long-term outcomes, but morbid obesity may adversely affect these outcomes. There is no proven benefit of aggressively treating obesity in the decompensated patient being considered for LT unless BMI is precluding a patient from being listed. The backbone of obesity treatment should be diet and exercise, being careful not to precipitate or worsen frailty and sarcopenia. There are limited data to support the use of pharmacologic treatments. BS is an alternative for treatment of obesity, and the ideal timing regarding LT is still unknown; SG is probably the procedure that has the best evidence in LT because it offers a good balance between safety and efficacy.

## Figures and Tables

**Table 1:** Timing of bariatric surgery in the liver transplant setting.

	PRE	DURING	POST
**PROS**	-Potential for improvement of liver function and delisting -Potential for decreasing risk of post-LT complications associated with obesity -Weight loss in order to achieve a certain BMI in centers where obesity is a contraindication for LT	-Single intervention and single recovery phase -Less risk of perioperative complications associated with portal hypertension	-Patient is more stable and without portal hypertension
**CONS**	-Potential for increased morbidity and mortality in patients with advanced cirrhosis	-Potential increased risk of staple line complications due to high dose steroids -Rapid weight loss may complicate immunosuppression dosing -May worsen intolerance to oral intake in the immediate postoperative period -Increased surgical time -Potential for increased rate of perioperative complications when compared to LT-only procedure -May worsen accelerated loss of bone mass in the first months after LT -May be cumbersome to the patient to learn post-LT care plus post-BS care	-Technically more challenging surgery because of post-LT adhesions -Increased infection risk due to immunosuppression -Steroids can interfere with healing

Based on information from Sharpton [59], García-Sesma [60], Diwan [53]. LT: Liver transplant; BMI: Body mass index; BS: Bariatric surgery.

**Table 2:** Pros, cons, and weight loss of different bariatric approaches in the liver transplant setting.

	Gastric Bypass	Sleeve Gastrectomy	Banding	Intragastric Balloon
**PROS**	-The most efficient in terms of weight loss	-Does not cause malabsorption, less risk for malnutrition -Less operative time, reducing anesthesia duration -Technically easier -Does not modify pharmacokinetics of tacrolimus or MMF	-The least invasive, requires minimal dissection -Technically speaking is the easiest of the surgical procedures	-Minimally invasive -Can potentially be used in the decompensated patient -Easiest of all the procedures
**CONS**	-No easy access to the biliary tract or the remnant stomach which may develop variceal bleeding -Potential to lead to malabsorption and undernutrition -Affects the PKs of immunosuppressants -Use of steroids may increase the risk of marginal ulcers	-Risk of perioperative bleeding if there are gastric varices -Risk of bleeding or leakage from staple line	-Risk of complications related to the band (infection, migration) -The least effective in terms of weight loss	-Contraindicated in patients with large esophageal varices, gastric varices, or severe portal gastropathy

MMF: Mycophenolate mofetil; PKs: Pharmacokinetics.

## Abbreviations

LT	Liver transplant
NAFLD	Non-alcoholic fatty liver disease
HCC	Hepatocellular carcinoma
ELTR	European Liver Transplant Registry
OPTN	Organ Procurement and Transplantation Network
SRTR	Scientific Registry of Transplant Recipients
BMI	Body mass index
LDLT	Living donor liver transplant
GWRW	Graft weight/recipient weight
MELDa	Model for End-Stage Liver Disease
HVPG	Hepatic venous pressure gradient
ICU	Intensive care unit
BS	Bariatric surgery
RYGB	Roux-en-Y gastric bypass
LAGB	Laparoscopic adjustable gastric banding
SG	Sleeve gastrectomy
CSPH	Clinically significant portal hypertension
MMF	Mycophenolate mofetil
PKs	Pharmacokinetics
